# Remedy for Dysmenorrhœa

**Published:** 1876-08

**Authors:** 


Remedy for Dysmenorrhœa and Consequent Sterility.—The following was published in the New Orleans *Medical News* nearly twenty years ago; it is republished at the request of a physician who has used it successfully in his practice: Dr. E. D. Fenner states that he has used for some years, in the treatment of dysmenorrhœa, with great success, the following mixture, originally recommended by Dr. Falk, of London: Take of gum guaiac, 1 ounce; balsam canadense, 1 ounce; ol. sassafras, 2 drams; merc. corrosiv. sublimat., 1 scruple; rect. spt. vini (alcohol), 8 ounces. "Dissolve the guaiac and balsam in one-half the spirit, and the corrosive sublimate in the other. Let the guaiac and balsam digest for several days; then pour off the clear liquor, mix with the sublimate, and add the oil. Dose—ten or twenty drops night and morning, in a glass of wine or water, *pro re nata.*" This was called by Dr. Falk "*Tinctura Antacrida.*"

Dr. Fenner says that he usually directs the patient to begin a day or two before the expected period, and take twenty-five drops in an infusion of sage or sweetened water, night and morning, until the discharge is freely established; then cease till the next period. In obstinate and severe cases the medicine should be commenced a week or ten days before the period, and if the pain appears the medicine should be taken every four or six hours till relieved. The pain usually disappears as soon as the discharge becomes free, but in most cases the discharge comes on without pain after taking a few doses. "I have known immediate relief to be given by a single dose taken in the paroxysm; but I have seen cases in which the pain was excruciating, causing shrieks and even violent convulsions. In such I have had to resort to a more prompt and efficient anæsthetic, as the inhalation of chloroform, or the following, which I have often known to act like a charm: Take of spirit camphor, 3 drams; chloroform, 2 drams; tinct. opii, 1 dram; mix; dose—a tea-spoonful in sweetened water once an hour till relieved. In violent hysterical spasms there is nothing comparable to the inhalation of chloroform. In the treatment of dysmenorrhœa it is important to obviate costiveness by the use of aloetic pills. When dysmenorrhœa is relieved by this treatment, conception almost invariably soon occurs in married women."—*Louisville Med. News.*

